# Association of the HLA–DRB1 gene with premature death, particularly from cardiovascular disease, in patients with rheumatoid arthritis and inflammatory polyarthritis

**DOI:** 10.1002/art.23149

**Published:** 2008-02

**Authors:** Tracey M Farragher, Nicola J Goodson, Haris Naseem, Alan J Silman, Wendy Thomson, Deborah Symmons, Anne Barton

**Affiliations:** 1 University of ManchesterManchester, UK; 2 University Hospital Aintree, Liverpool UniversityLiverpool, UK

## Abstract

**Objective:**

To examine the role of the variants of the *PTPN22* and *HLA*–*DRB1* genes as predictors of mortality in inflammatory polyarthritis (IP) and rheumatoid arthritis (RA).

**Methods:**

Patients were recruited from a primary care–based inception cohort of patients with IP and were followed up prospectively. For patients who died, the cause and date of death was obtained. Cox proportional hazards regression models were used to assess the association of the *HLA*–*DRB1* (including the shared epitope [SE]) and *PTPN22* genes with the risk of death from all causes and from cardiovascular disease (CVD) and to assess the interactions between SE, smoking, and anti–cyclic citrullinated peptide (anti-CCP) status, adjusted by age at symptom onset and sex.

**Results:**

DNA samples were available from 1,022 IP patients. During followup, 751 of them (74%) satisfied the American College of Rheumatology 1987 criteria for RA, and 242 of them (24%) died. Carriage of 2 copies of SE alleles predicted death from all causes (hazard ratio [HR] 1.57 [95% confidence interval (95% CI) 1.1–2.2]) and from CVD (HR 1.68 [95% CI 1.1–2.7]). This effect was most marked for individuals with the *HLA*–DRB1*01/*04 combination. An interaction of smoking, SE alleles, and anti-CCP antibodies was observed and was associated with the greatest risk of death from CVD (HR 7.81 [95% CI 2.6–23.2]). No association of the *PTPN22* gene with mortality was detected.

**Conclusion:**

SE alleles, particularly compound heterozygotes, are associated with death from all causes and from CVD, independently of autoantibody status. However, the combination of SE, smoking, and anti-CCP antibodies is associated with a high risk of premature death in patients with IP and RA, which raises the possibility of a targeted strategy to prevent CVD in these patients.

It is being increasingly recognized that people with rheumatoid arthritis (RA) are at greater risk of premature death as compared with the general population and that cardiovascular disease (CVD) is responsible for most of this excess mortality (for review, see ref.^1^). One hypothesis is that inflammation may promote atherosclerosis. Indeed, elevation of the C-reactive protein (CRP) level, a marker of systemic inflammation, has been shown to predict CVD in the general population (2). Previous studies in patients with RA have also confirmed that it is those with the most active inflammatory disease who carry the greatest increased risk of death from all causes and, in particular, death from CVD (3–6). However, increased mortality rates are not seen in all diseases with a high inflammatory burden, such as Crohn's disease, for example, suggesting that other factors also play a role. To explore other possible pathways, we investigated whether genetic variants associated with RA susceptibility and/or severity may also predict all-cause and CVD mortality in these patients.

The major susceptibility genes identified for both RA and inflammatory polyarthritis (IP) in populations of northern European descent are *HLA*–*DRB1* (7) and *PTPN22* (8). While investigations of the latter gene suggest that it plays a role in susceptibility, rather than outcome (9), the *HLA*–*DRB1* gene has been associated with disease severity in IP patients in general and in RA patients in particular (7,10,11). A group of *HLA*–*DRB1* alleles that share amino acid homology in the third hypervariable region of the DRβ chain, collectively referred to as the shared epitope (SE), are a broad genetic marker that has been associated with outcomes of RA, such as disability (10) and erosive disease (11,12). Other studies have identified specific *HLA*–*DRB1* genotypes that are associated with either severe RA or extraarticular manifestations of RA (13). For example, both *HLA*–DRB1*0404 and *HLA*–DRB1*0401 are associated with erosive disease (10), and homozygosity for the *HLA*–DRB1*0401 genotype has been associated with systemic organ involvement (14). Furthermore, the genotypes *HLA*–DRB1*0101/*0404, *0401/*0401, and *0401/*0404 have all been associated with vasculitis (15), while the latter genotype has also been associated with both Felty's syndrome (16) and early-onset aggressive RA in men (17).

The presence of SE alleles correlates with the presence of both rheumatoid factor (RF) (18) and anti–cyclic citrullinated peptide (anti-CCP) antibodies (19), and recent studies suggest that these autoantibodies, in particular, anti-CCP antibodies, are on the pathway by which SE leads to severe disease (19). Furthermore, it has been proposed that an interaction between smoking and the *HLA*–*DRB1* SE alleles may trigger the production of anti-CCP antibodies, and this may contribute to the development of RA (20). All 3 of these factors have also been shown to be independently associated with the severity and onset of RA (7,10–12,21–28).

There are, therefore, an increasing number of serologic and genetic markers that may be predictive of both the subsequent cumulative level of inflammation and the disease severity, which in turn, is associated with mortality. The aim of the present study was to examine whether either the *PTPN22* gene or genotypes of the *HLA*–*DRB1* gene, which are associated with disease severity, are predictors of all-cause and CVD mortality in patients with IP or RA and whether any effect is independent of autoantibody status. The study also aimed to examine whether the gene–environment interaction that results in susceptibility to RA could also predict subsequent mortality in IP.

## PATIENTS AND METHODS

### Study design

We undertook a cohort analysis comparing survival in patients with IP stratified by SE carrier status, *HLA*–*DRB1* genotype status, and PTPN22*1858T carrier status.

### Study population

Patients were recruited from the Norfolk Arthritis Register (NOAR), a primary care–based inception cohort of patients with recent onset IP. NOAR aims to recruit all adults ages 16 years and older who have swelling of at least 2 joints persisting for at least 4 weeks with symptom onset after January 1, 1989. NOAR covers the former Norwich Health Authority and is notified of cases via general practitioners or staff at hospitals within the catchment area (29).

Patients who were subsequently diagnosed by a hospital consultant as having a condition other than RA, IP, psoriatic arthritis, or postviral arthritis, were excluded from the study. Between 1989 and 1994, a total of 1,424 patients were referred to NOAR who satisfied the above criteria. For this investigation, consecutive patients recruited by NOAR within this time period for whom a DNA sample was available were studied (n = 1,022). All patients were white.

### Data collection

Data were collected by a research nurse via a structured interview and clinical examination shortly after registration (baseline). Demographic data collected at baseline included age at symptom onset, sex, smoking status, and time from symptom onset to presentation to NOAR. A further assessment was performed annually until the fifth year and then at the seventh and tenth years. At each assessment, standard clinical variables were collected (for details, see ref.^29^). A blood sample was taken for RF, anti-CCP, and CRP testing. RF was measured using a latex agglutination technique, where a titer ≥1:40 was classified as RF positive. Anti-CCP was tested using the Axis-Shield Diastat kit according to the manufacturer's instructions (Axis-Shield, Dundee, UK), where a concentration of >5 units/ml was classified as anti-CCP antibody positive. CRP levels (in mg/liter) were measured using an end-point immunoturbidimetric agglutination method. The Disease Activity Score 28-joint assessment (DAS28) was calculated from the tender and swollen joint counts in 28 joints and the CRP value (for calculation methods, see http://www.das-score.nl/www.das-score.nl/index.html). Functional disability was assessed at baseline by having the patients complete the Health Assessment Questionnaire (HAQ), as modified for use in British patients (30). The American College of Rheumatology (ACR; formerly, the American Rheumatism Association) 1987 criteria for the classification of RA (31) were applied both cross-sectionally at baseline and cumulatively. For the purposes of this analysis, patients were deemed to have RA if they satisfied the ACR criteria by the tenth-year assessment or by the time of death, whichever was sooner.

### *HLA*–*DRB1* genotyping

*HLA* genotyping was performed as described previously (7). Subtyping at the *HLA*–*DRB1* locus was performed to identify the presence of the SE, which was defined as the presence of any of the following alleles: *HLA*–DRB1*0101, *0102, *0104, *0401, *0404, *0405, *0408, or *1001.

### *PTPN22* genotyping

Genotyping of the functional polymorphism PTPN22*C1858T (rs2476601) was undertaken using the Sequenom platform (Sequenom, San Diego, CA) according to the manufacturer's instructions (available at http://www.sequenom.com).

### Ascertainment of deaths

The Office for National Statistics (ONS) provided details of the patients' deaths, including the cause and date of death. The cause of death was coded using the International Classification of Diseases, Ninth Revision (ICD-9) (32) until the end of 2000, and then the Tenth Revision (ICD-10) from 2001 onward (33). For this analysis, all codes were converted to ICD-10 codes, and the underlying causes of death were grouped by ICD-10 chapter. Each patient was followed up from the time of disease symptom onset until death or until December 31, 2005, whichever was sooner. If a patient moved from the UK or was no longer registered with a general practitioner, the ONS notified NOAR of the date of “embarkation.” For these patients, followup was censored to the time of embarkation.

### Statistical analysis

The association of *HLA*–*DRB1* genotypes and the PTPN22*1858T risk allele carriage with mortality risk was assessed using Cox proportional hazards regression models after adjusting for age at symptom onset and sex. The interaction between baseline RF status, anti-CCP status, and *HLA*–*DRB1* genotypes and PTPN22*1858T allele carriage was assessed via Cox proportional hazards regression models after adjusting for age at onset and sex. A similar analysis was undertaken to assess whether associations with genotypes were independent of the CRP value at baseline, with patients divided according to whether the CRP was ≤10 mg/dl or >10 mg/dl.

Causes of death were determined from the death certificate. Analyses were undertaken for death from all causes as well as for death from CVD. Death from CVD was recorded in 2 ways: those for whom a cardiovascular condition (ICD-10 codes I00–I99) was recorded as the main underlying cause of death and those for whom a cardiovascular condition was mentioned anywhere on the death certificate.

Finally the interactions between SE status (0, 1, or 2 SE alleles), smoking status at baseline (never, previous, or current smoker), baseline anti-CCP status (negative or positive), and mortality risk (all causes and CVD) were explored through Cox proportional hazards regression models, adjusted by age at symptom onset and sex. All analyses were performed using the whole IP cohort, while the analysis between genotypes and all-cause mortality was also performed in the subset who met the ACR criteria for RA by 10 years or by the time of death, if sooner. All statistical analyses were conducted using Stata version 9 software (34).

## RESULTS

### Baseline characteristics of the study population

A DNA sample was available for 1,022 IP patients, 751 of whom satisfied the ACR criteria for RA by 10 years or by their time of death, if sooner. Their baseline characteristics are shown in Table 1. As expected, markers of disease severity, such as the DAS28 score, CRP level, and the HAQ score, were higher in the subgroups of patients with ACR-classified RA, the RF-positive subgroup, and the anti-CCP–positive subgroup at baseline.

**Table 1:** Clinical and demographic characteristics of the entire IP cohort and the RA, RF+, and anti-CCP+ subgroups*

Characteristic	Entire IP cohort (n = 1,022)	RA subgroup (met ACR criteria by the tenth year) (n = 751)	IP cohort patients RF+ at baseline (n = 275)	IP cohort patients anti-CCP+ at baseline (n = 204)
Age at symptom onset, median (IQR) years	54 (41–66)	55 (44–67)	57 (47–67)	57 (48–65)
No. (%) female	661 (64.7)	499 (66.4)	162 (58.9)	113 (55.4)
Symptom duration at registration, median (IQR) months	5 (2–12)	5 (2–12)	5 (2–12)	7 (3–14)
No. (%) RF positive (titer ≥1:40)†				
At baseline	275 (29.1)	247 (35.7)	275 (100)	141 (69.1)
By the tenth year	420 (41.2)	380 (50.7)	275 (100)	176 (86.3)
No. (%) meeting ACR criteria for RA				
At baseline	472 (46.2)	472 (62.9)	197 (71.6)	136 (66.7)
By the tenth year	751 (73.5)	751 (100)	247 (89.8)	186 (91.2)
CRP at baseline, median (IQR) mg/dl‡	5 (0–15)	7 (1–19)	9 (2–23)	12 (4–32)
DAS28 at baseline, mean (95% CI)	3.95 (3.86–4.05)	4.38 (4.28–4.48)	4.25 (4.07–4.43)	4.39 (4.20–4.59)
No. (%) with anti-CCP antibodies at baseline§	204 (27.6)	186 (35.7)	141 (70.2)	204 (100)
HAQ score at baseline, median (IQR)¶	0.75 (0.25–1.38)	0.88 (0.38–1.63)	0.75 (0.38–1.63)	0.88 (0.38–1.63)
No. of swollen and tender joints at baseline, median (IQR)	3 (0–8)	4 (1–10)	3 (1–9)	4 (1–9)
Smoking status at baseline, no. (%)#				
Never smoked	314 (31.2)	240 (32.4)	64 (23.6)	46 (22.9)
Ex-smoker ≥10 years	266 (26.4)	194 (26.2)	59 (21.8)	40 (19.9)
Ex-smoker <10 years	158 (15.7)	115 (15.5)	53 (19.6)	43 (21.4)
Current smoker	268 (26.6)	192 (25.9)	95 (35.1)	72 (35.8)

* IP = inflammatory polyarthritis; RA = rheumatoid arthritis; ACR = American College of Rheumatology; IQR = interquartile range; DAS28 = Disease Activity Score 28-joint assessment; 95% CI = 95% confidence interval.

† Rheumatoid factor (RF) was measured in 946 patients at baseline and in 1,019 patients by the tenth year.

‡ C-reactive protein (CRP) levels were measured in 858 patients at baseline.

§ Anti–cyclic citrullinated peptide (anti-CCP) antibodies were measured in 740 patients at baseline.

¶ Health Assessment Questionnaire (HAQ) scores were determined in 1,011 patients at baseline.

# Smoking status was determined in 1,006 patients at baseline.

An increasing frequency of SE allele carriage was observed as the stringency of phenotype definition increased. Restricting the analysis to the subgroup with ACR-classified RA, and then to the RF-positive subgroup, and finally, to the anti-CCP–positive subgroup yielded increasing frequencies of patients with SE alleles (Table 2). In contrast, only slight differences in frequencies of carriage of the PTPN22*1658T susceptibility allele were noted between these subgroups (Table 2).

**Table 2:** *HLA*–*DRB1* and *PTPN22* genotypes in the entire IP cohort and in the RA, RF+, and anti-CCP+ subgroups*

*HLA*–*DRB1* and *PTPN22* groups	No. (%) of entire IP cohort (n = 1,022)	No. (%) of RA subgroup (met ACR criteria by the tenth year) (n = 751)	No. (%) of IP cohort RF+ at baseline (n = 275)	No. (%) of IP cohort anti-CCP+ at baseline (n = 204)
0 copies of SE alleles	416 (40.7)	288 (38.3)	81 (29.5)	40 (19.6)
1 copy of SE alleles	460 (45.0)	349 (46.5)	133 (48.4)	108 (52.9)
*HLA*–DRB1*01	167 (16.3)	119 (15.8)	43 (15.6)	28 (13.7)
*HLA*–DRB1*04	281 (27.5)	220 (29.3)	87 (31.6)	76 (37.3)
*HLA*–DRB1*10	12 (1.2)	10 (1.3)	3 (1.1)	4 (2.0)
2 copies of SE alleles	146 (14.3)	114 (15.2)	61 (22.2)	56 (27.5)
*HLA*–DRB1*01/*01	15 (1.5)	11 (1.5)	2 (0.7)	0
*HLA*–DRB1*01/*04	59 (5.8)	43 (5.7)	23 (8.4)	18 (8.8)
*HLA*–DRB1*0101/*0401	36 (3.5)	24 (3.2)	13 (4.7)	11 (5.4)
*HLA*–DRB1*0101/*0404	12 (1.2)	10 (1.3)	5 (1.8)	4 (2.0)
*HLA*–DRB1*01/*10	3 (0.3)	3 (0.4)	0	0
*HLA*–DRB1*04/*04	63 (6.2)	52 (6.9)	34 (12.4)	37 (18.1)
*HLA*–DRB1*0401/*0401	16 (1.6)	14 (1.9)	7 (2.5)	7 (3.4)
*HLA*–DRB1*0401/*0404	33 (3.2)	26 (3.5)	22 (8.0)	22 (10.8)
*HLA*–DRB1*04/*10	6 (0.6)	5 (0.7)	2 (0.7)	1 (0.5)
PTPN22*1858T allele				
Negative	608 (77.1)	435 (77.5)	138 (73.8)	121 (72.5)
Positive	181 (22.9)	126 (22.5)	49 (26.2)	46 (27.5)

* Common genotype combinations are shown below the broad subtype combinations. IP = inflammatory polyarthritis; RA = rheumatoid arthritis; RF = rheumatoid factor; anti-CCP = anti–cyclic citrullinated peptide; ACR = American College of Rheumatology; SE = shared epitope.

### Role of CRP, RF, anti-CCP, and smoking status at baseline in predicting mortality

By December 31, 2005, a total of 242 patients (23.7%) had died. CRP levels and smoking status at baseline were predictors of death from all causes (Table 3). While ex-smokers did not have an increased risk of death (hazard ratio [HR] 1.02 [95% confidence interval (95% CI) 0.73–1.43]), compared with those who never smoked, current smokers did have a higher mortality risk (HR 1.43 [95% CI 0.97–2.11]), although the difference was not statistically significant. When the analysis was restricted to those who satisfied the ACR criteria for RA by 10 years, current smokers did have a significantly higher mortality risk compared with nonsmokers (HR 1.54 [95% CI 1.00–2.38]). The duration of smoking did not appear to have an influence on mortality risk. Compared with those who had never smoked or those who were ex-smokers, the estimates of the risk of mortality in smokers did not increase with the number of years smoked (categorized by tertiles). Information on the number of cigarettes smoked per day was not collected.

**Table 3:** Risk of death from all causes in the entire IP cohort and in the RA subgroup, by clinical characteristics and *HLA*–*DRB1* and *PTPN22* genotypes*

	Entire IP cohort (n = 1,022)		RA subgroup (met ACR criteria by the tenth year) (n = 751)	
Group	No. of patients	HR (95% CI)	No. of patients	HR (95% CI)
CRP at baseline				
≤10 mg/dl (comparator)	580	1.00	382	1.00
>10 mg/dl	278	1.56 (1.19–2.06)	242	1.66 (1.22–2.26)
Smoking status at baseline				
Never smoked (comparator)	314	1.00	240	1.00
Ex-smoker	424	1.02 (0.73–1.43)	309	1.07 (0.73–1.56)
Current smoker	268	1.43 (0.97–2.11)	192	1.54 (1.00–2.38)
Never smoked or ex-smoker at baseline (comparator)	738	1.00	549	1.00
First tertile of smoking duration at baseline (0–25 years)	95	1.41 (0.50–3.96)	61	1.45 (0.45–4.71)
Second tertile of smoking duration at baseline (26–39 years)	84	1.53 (0.80–2.93)	59	1.43 (0.64–3.19)
Third tertile of smoking duration at baseline (40–68 years)	87	1.39 (0.99–1.94)	70	1.49 (1.03–2.15)
RF titer at baseline				
<1:40 (comparator)	671	1.00	445	1.00
≥1:40	275	1.61 (1.24–2.1)	247	1.74 (1.30–2.34)
Anti-CCP antibodies at baseline				
No (comparator)	536	1.00	335	1.00
Yes	204	1.37 (1.00–1.88)	186	1.50 (1.04–2.15)
SE alleles				
0 copies of SE alleles (comparator)	416	1.00	288	1.00
1 copy of SE alleles	460	0.98 (0.74–1.30)	349	0.96 (0.70–1.32)
2 copies of SE alleles	146	1.55 (1.08–2.24)	114	1.54 (1.03–2.31)
0 or 1 SE allele (comparator)	876	1.00	637	1.00
2 SE alleles	146	1.57 (1.13–2.19)	114	1.58 (1.10–2.27)
0 or 1 SE allele (comparator)	876	1.00	637	1.00
*HLA*–DRB1*01/*01	15	1.89 (0.70–5.11)	11	1.74 (0.55–5.48)
*HLA*–DRB1*01/*04	59	1.99 (1.24–3.19)	43	2.25 (1.34–3.79)
*HLA*–DRB1*0101/*0401	36	1.66 (0.94–2.92)	24	1.72 (0.90–3.27)
*HLA*–DRB1*0101/*0404	12	3.00 (0.94–9.52)	10	3.30 (1.03–10.57)
*HLA*–DRB1*04/*04	63	1.26 (0.75–2.09)	52	1.15 (0.65–2.03)
*HLA*–DRB1*0401/*0401	16	2.63 (1.22–5.64)	14	2.38 (1.04–5.45)
*HLA*–DRB1*0401/*0404	33	0.96 (0.47–1.96)	26	0.83 (0.36–1.88)
PTPN22*1858T allele				
Negative (comparator)	608	1.00	435	1.00
Positive	181	1.07 (0.73–1.55)	126	0.76 (0.47–1.21)

* Data were adjusted for age at symptom onset and sex. IP = inflammatory polyarthritis; RA = rheumatoid arthritis; ACR = American College of Rheumatology; HR = hazard ratio; 95% CI = 95% confidence interval; CRP = C-reactive protein; RF = rheumatoid factor; anti-CCP = anti–cyclic citrullinated peptide; SE = shared epitope.

The RF status and the anti-CCP status at baseline were also predictors of death from all causes (Table 3). For example, in the IP group as a whole, the presence of anti-CCP antibodies at baseline was a significant predictor of mortality risk (HR 1.37 [95% CI 1.00–1.88]) as compared with the absence of anti-CCP antibodies at baseline. The predictive value of both RF positivity and presence of anti-CCP antibodies at baseline improved when restricted to those who met the ACR criteria for RA by 10 years (Table 3). However, having both anti-CCP antibodies and RF at baseline did not confer a higher mortality risk than having just 1 of these markers, as compared with those who were negative for both markers (HR 1.47 [95% CI 1.02–2.11] in the IP cohort and HR 1.65 [95% CI 1.09–2.51] in the RA subgroup).

### *HLA*–*DRB1* and *PTPN22* as predictors of death from all causes

The influence of *HLA*–*DRB1* and PTPN22*1858T phenotypes on mortality risk was similar in the entire IP cohort and in the subgroup who satisfied the ACR criteria for RA by 10 years (Table 3). Compared with carriage of 0 SE alleles, those with 2 copies of SE alleles had an increased risk of death from all causes, both in the whole IP cohort (HR 1.55 [95% CI 1.08–2.24]) and in the subgroup with ACR-classified RA (HR 1.54 [95% CI 1.03–2.31]) (Table 3). There was no increased risk in those with only 1 SE allele.

When looking at the broad groups (Table 3), patients with an *HLA*–DRB1*01/*04 combination had double the mortality risk compared with those carrying 0 or 1 SE allele (HR 1.99 [95% CI 1.24–3.19] in the entire IP cohort). In order to explore this further, we undertook a more detailed genotype analysis. The frequency of the common genotype combinations in the 146 patients who carried 2 SE alleles is shown in Table 2 and the resultant HRs in Table 3. Carriage of *HLA*–DRB1*0101/*0404 was associated with the highest mortality risk; this was most marked in those who satisfied the ACR criteria for RA by 10 years (HR 3.30 [95% CI 1.03–10.57]). Furthermore, carriage of *HLA*–DRB1*0401/*0401* was also associated with an increased mortality risk in the entire IP cohort (HR 2.63 [95% CI 1.22–5.64]).

No association between PTPN22*1858T allele carriage and the risk of death from all causes was detected.

### *HLA*–*DRB1* and *PTPN22* as predictors of death from CVD

CVD was the main cause of death in 76 of the 242 patients who died (31.4%), while in a further 48 patients (overall total of 51.2%), CVD was mentioned elsewhere on the death certificate. The majority of *HLA*–*DRB1* genotype combinations found to be predictors of death from all causes were also predictors of death from CVD, and the risk of death was higher for CVD than for all causes (Table 4). For example, compared with patients who had 0 or 1 SE allele, the CVD mortality risk in those carrying the *HLA*–DRB1*01/*04 combination was increased over 3-fold (HR 3.03 [95% CI 1.37–6.68]) (Table 4). Carriage of the DRB1*0101/*0401 genotype was associated with the highest significant risk of death (HR 3.58 [95% CI 1.52–8.47] in the IP cohort). Patients with 1 copy of the *HLA*–DRB1*0401 allele had a higher mortality risk, although it did not reach statistical significance (HR 4.04 [95% CI 0.97–16.87]). As with all-cause mortality, no relationship between *PTPN22* susceptibility allele carriage and CVD mortality was noted.

**Table 4:** Risk of death from CVD in patients with a cardiovascular condition listed as the main cause of death or mentioned anywhere on the death certificate, by *HLA*–*DRB1* genotype*

		HR (95% CI) for cardiovascular condition	
*HLA*–*DRB1* group	No. of patients	As main cause of death (n = 76)	Mentioned anywhere on death certificate (n = 124)
0 copies of SE alleles (comparator)	416	1.00	1.00
1 copy of SE alleles	460	0.87 (0.53–1.43)	1.02 (0.69–1.51)
2 copies of SE alleles	146	1.65 (0.86–3.16)	1.70 (1.02–2.83)
0 or 1 SE allele (comparator)	876	1.00	1.00
2 SE alleles	146	1.77 (0.97–3.23)	1.68 (1.06–2.67)
0 or 1 SE allele (comparator)	876	1.00	1.00
*HLA*–DRB1*01/*01	15	†	1.20 (0.17–8.66)
*HLA*–DRB1*01/*04	59	3.03 (1.37–6.68)	2.18 (1.19–3.99)
*HLA*–DRB1*0101/*0401	36	3.58 (1.52–8.47)	2.13 (1.10–4.14)
*HLA*–DRB1*0101/*0404	12	2.67 (0.36–19.78)	2.03 (0.28–14.82)
*HLA*–DRB1*04/*04	63	1.21 (0.48–3.01)	1.17 (0.54–2.53)
*HLA*–DRB1*0401/*0401	16	4.04 (0.97–16.87)	2.91 (0.71–11.97)
*HLA*–DRB1*0401/*0404	33	0.95 (0.30–3.04)	1.12 (0.45–2.75)

* Data were adjusted for age at symptom onset and sex. CVD = cardiovascular disease; HR = hazard ratio; 95% CI = 95% confidence interval; SE = shared epitope.

† Too few deaths to provide robust hazard ratio estimates.

### *HLA*–*DRB1* and *PTPN22* as predictors of death in patients positive/negative for RF and anti-CCP

An association between the *HLA*–DRB1*01/*04 combination and death was seen in patients who were RF-positive and in those who were RF-negative at baseline, although the association was not significant in the RF-negative group (Table 5). Compared with RF-negative patients with 0 or 1 SE allele, RF-positive patients with 0 or 1 SE allele had a higher mortality risk (HR 1.61 [95% CI 1.21–2.15]), and in those with 2 SE alleles, mortality risk was higher still (HR 1.97 [95% CI 1.23–3.17]). However, the interaction term between RF status at baseline and the presence of 0 or 1 versus 2 copies of SE alleles was not significant (*P* = 0.67). Again, it was a combination of *HLA*–DRB1*01/*04 that gave the greatest hazard ratios in the RF-negative individuals (HR 1.90 [95% CI 0.99–3.63]).

**Table 5:** Risk of death from all causes, by *HLA*–*DRB1* and *PTPN22* genotypes, in subgroups of patients with and without RF or anti-CCP at baseline*

	RF status at baseline (n = 946)				Anti-CCP status at baseline (n = 858)			
	Negative		Positive		Negative		Positive	
*HLA*–*DRB1* and *PTPN22* groups	No. of patients	HR (95% CI)	No. of patients	HR (95% CI)	No. of patients	HR (95% CI)	No. of patients	HR (95% CI)
0 copies of SE alleles (comparator)	305	1.00	81	1.90 (1.23–2.93)	261	1.00	40	1.35 (0.74–2.46)
1 copy of SE alleles	293	0.97 (0.67–1.39)	133	1.42 (0.95–2.10)	229	0.78 (0.53–1.16)	108	1.03 (0.66–1.61)
2 copies of SE alleles	73	1.41 (0.81–2.46)	61	1.94 (1.17–3.24)	46	0.86 (0.39–1.91)	56	1.57 (0.896–2.77)
0 or 1 SE allele (comparator)	598	1.00	214	1.61 (1.21–2.15)	490	1.00	148	1.26 (0.89–1.80)
2 SE alleles	73	1.43 (0.85–2.42)	61	1.97 (1.23–3.17)	46	0.99 (0.46–2.13)	56	1.79 (1.05–3.04)
0 or 1 SE allele (comparator)	598	1.00	214	1.61 (1.21–2.15)	490	1.00	148	1.26 (0.89–1.80)
*HLA*–DRB1*01/*01	10	1.99 (0.49–8.09)	2	†	8	1.20 (0.17–8.67)	0	–
*HLA*–DRB1*01/*04	33	1.90 (0.99–3.63)	23	3.07 (1.49–6.31)	23	1.05 (0.42–2.59)	18	2.73 (1.18–6.29)
*HLA*–DRB1*0101/*0401	20	1.79 (0.87–3.7)	13	2.13 (0.79–5.79)	13	0.99 (0.36–2.72)	11	2.60 (0.95–7.10)
*HLA*–DRB1*0101/*0404	7	2.44 (0.34–17.63)	5	4.15 (1.01–17.04)	5	2.89 (0.40–21.08)	4	2.17 (0.30–15.72)
*HLA*–DRB1*04/*04	25	0.77 (0.24–2.42)	34	1.65 (0.89–3.07)	13	1.12 (0.15–8.06)	37	1.44 (0.72–2.86)
*HLA*–DRB1*0401/*0401	7	1.00 (0.14–7.22)	7	3.08 (1.13–8.42)	5	1.59 (0.22–11.53)	7	2.82 (0.69–11.54)
*HLA*–DRB1*0401/*0404	9	0.45 (0.06–3.23)	22	1.57 (0.73–3.37)	4	†	22	1.42 (0.62–3.25)
PTPN22*1858T allele								
Negative (comparator)	418	1.00	138	1.50 (1.02–2.21)	369	1.00	121	1.54 (1.0–2.37)
Positive	120	1.34 (0.85–2.12)	49	1.14 (0.60–2.16)	106	1.25 (0.77–2.05)	46	1.23 (0.63–2.39)

* Data were adjusted for age at symptom onset and sex. RF = rheumatoid factor; anti-CCP = anti–cyclic citrullinated peptide; HR = hazard ratio; 95% CI = 95% confidence interval; SE = shared epitope.

† Too few deaths to provide robust hazard ratio estimates.

The association between the *HLA*–DRB1*01/*04 combination and mortality risk was independent of the baseline CRP value (data not shown), but the baseline CRP value alone was a good predictor of subsequent death. Compared with patients whose CRP level was ≤10 mg/liter at baseline and who carried 0 or 1 SE allele, there was a higher mortality risk in patients whose CRP level was >10 mg/liter and who carried 0 or 1 SE allele (HR 1.54 [95% CI 1.14–2.07]) or who carried 2 SE alleles (HR 2.16 [95% CI 1.29–3.63]).

In contrast, baseline anti-CCP antibody status showed a significant association with mortality risk only when 2 copies of the SE alleles were also present (Table 5).

### Interaction between SE alleles, smoking status, and anti-CCP antibody status as a predictor of death

Complete information on smoking and anti-CCP antibody status at baseline was available for 728 of the IP patients. The presence of anti-CCP antibodies at baseline was associated with premature death from CVD even in nonsmokers with 0 or 1 copy of the SE alleles (HR 2.92 [95% CI 0.99–8.65]), as well as in current smokers (HR 2.80 [95% CI 1.13–6.94]) (Table 6). The highest risk of death from all causes was in patients who at baseline were current smokers, had anti-CCP antibodies, and carried 2 SE alleles (HR 3.57 [95% CI 1.34–9.50]). The risk of death from CVD was substantially higher in this group of patients (HR 7.81 [95% CI 2.63–23.22]). Furthermore, the interaction term between current smoking, anti-CCP antibody positivity, and carriage of 2 copies of SE alleles was significant for both all-cause (*P* = 0.03) and CVD (*P* = 0.02) mortality.

**Table 6:** Risk of death from all causes and from CVD, by anti-CCP status, in the entire IP cohort (n = 728) categorized by SE and smoking status*

	0 or 1 SE allele (n = 627)			2 SE alleles (n = 101)		
Anti-CCP group	Never smoked	Ex-smoker	Current smoker	Never smoked	Ex-smoker	Current smoker
Anti-CCP, no. of patients						
Absent	162	202	117	16	15	15
Present	31	57	58	15	26	14
Death from all causes, HR (95% CI)						
Anti-CCP						
No	1.00 (comparator)	1.08 (0.65–1.81)	1.30 (0.68–2.49)	2.55 (0.76–8.57)	0.94 (0.22–4.02)	0.63 (0.14–2.75)
Yes	1.51 (0.67–3.4)	1.02 (0.53–1.94)	2.03 (1.06–3.86)	0.76 (0.18–3.24)	2.12 (0.93–4.83)	3.57 (1.34–9.50)
Death from CVD, HR (95% CI)						
Anti-CCP						
No	1.00 (comparator)	1.35 (0.65–2.81)	1.09 (0.39–3.06)	2.85 (0.62–13.21)	1.80 (0.39–8.24)	0.54 (0.07–4.44)
Yes	2.92 (0.99–8.65)	1.49 (0.64–3.47)	2.80 (1.13–6.94)	0.73 (0.09–5.70)	2.08 (0.57–7.62)	7.81 (2.63–23.22)

* Only inflammatory polyarthritis (IP) patients with complete information on smoking and anti–cyclic citrullinated peptide (anti-CCP) antibody status at baseline were included in the analysis. Data were adjusted for age at symptom onset and sex. CVD = cardiovascular disease; SE = shared epitope; HR = hazard ratio; 95% CI = 95% confidence interval.

### Calculations of study power

Power calculations were undertaken based on sample sizes used in the all-cause mortality analysis between smoking status, anti-CCP antibody status, and carriage of 2 copies of SE alleles, since this analysis had the smallest numbers of patients and events. There was >70% power to detect a statistically significant difference at the 5% level for most of the subgroups shown in Table 6, as compared with the reference group. The power was lower for the 2 subgroups that actually showed significant mortality risk: those who were current smokers and anti-CCP positive and had 0 or 1 SE allele (power 49.5%) or had 2 SE alleles (power 56.8%).

## DISCUSSION

This study is the first to show that *HLA*–*DRB1* genotype predicts both all-cause and CVD mortality in patients with IP recruited from primary care settings. The findings were similar in the IP group as a whole and in the subgroup who met the ACR criteria for RA. The presence of 2 copies of SE alleles, in particular, the *HLA*–DRB1*01/*04 combination and homozygosity for the *HLA*–DRB1*0401 alleles, was associated with high hazard ratios for mortality. The effect sizes were higher for death from CVD than for death from all causes. No association between carriage of PTPN22*1858T susceptibility alleles and either all-cause mortality or CVD mortality was demonstrated.

The mechanism by which SE homozygosity promotes increased mortality rates is not clear, although previous studies have shown that compound heterozygosity is associated with more severe disease, extraarticular manifestations, and vasculitis in RA patients (11,14,15). We have shown that the combination of *HLA*–DRB1*04 with *HLA*–DRB1*01 is associated with death from CVD and from all causes. This particular SE genotype has previously been associated with radiographic progression (35) and an increased requirement for joint replacement (14). Such outcomes reflect more severe disease, and therefore, our findings support the hypothesis that it is the increased inflammatory disease burden that promotes premature death in patients with IP. Although the baseline CRP level was predictive of both all-cause and CVD mortality, there was an additional effect conferred by carriage of 2 copies of SE alleles.

Our findings are consistent with the results of a previous investigation of associations between *HLA*–*DRB1* genotype combinations and endothelial dysfunction, a critical early step in the development of atheroma (36). In that study, 2 copies of SE alleles, as compared with 0 or 1 copy, and the presence of the *HLA*–DRB1*0404 SE allele in particular was associated with endothelium-dependent vasodilatation.

Other explanations for our findings can be considered. It is possible that certain *HLA* genotypes may promote the production of specific T cells, and inheritance of 2 RA-associated *HLA*–*DRB1* alleles may increase the production of these cells (37). The T cell repertoire in patients with RA shows less diversity, with the resultant emergence of dominant epitopes. For example, CD8+ large granular lymphocytes as well as CD4+,CD28^null^ T cells are more common in RA patients, particularly those with extraarticular disease, and the latter T cells are also found in patients without RA who have acute coronary syndromes (13,37). It is possible that these T cells are directly involved in vascular wall damage. Both activated T cells and T cell lymphokines have been identified in atherosclerotic plaques, and furthermore, expression of class II major histocompatibility complex molecules by smooth muscle cells on the atherosclerotic intima, but not in the normal artery, has been demonstrated (38). These findings support the hypothesis that the SE may promote death from CVD by enabling T cell–mediated vascular damage and inflammation.

If the latter scenario were true, one might expect to find evidence of a link between *HLA*–*DRB1* variants and CVD mortality rates in the general population, since SE homozygosity occurs at a reasonable frequency (7). However, previous whole genome scans of families with coronary artery disease have revealed no evidence of linkage to the *HLA* region (39,40). An association has been observed between lipoprotein(a) levels, infection, and certain *HLA*–*DRB1* genotypes (41), but another small study found no association between *HLA*–*DRB1* genotypes and coronary artery disease (38).

Recent reports have proposed that possession of the SE predisposes to both the susceptibility to IP and the severity of IP, and RA in particular, by creating a permissive environment for the production of anti-CCP antibodies (42). This idea is based first on studies showing that anti-CCP antibodies correlate better with disease severity markers than with the presence of SE alleles. Second, citrullinated peptides are bound with higher affinity to SE alleles, and this, in turn, is associated with increased activation of CD4+ T cells (43). These findings raise the question of whether the primary association with death from CVD may be with the anti-CCP antibody status. Indeed, seropositivity for RF (highly correlated with the presence of anti-CCP antibodies in RA patients) in patients without arthritis has been reported to be associated with death from CVD (44). However, a recent cross-sectional study of RA patients found that RF, but not anti-CCP antibodies, was associated with mortality (45). In the current prospective study, we confirmed that both RF and anti-CCP antibodies are associated with premature death. However, the risk of premature death was increased when anti-CCP antibodies were present in patients with a background of smoking and carriage of 2 copies of SE alleles. Thus, in addition to increasing susceptibility to RA, we have now shown that this combination is also associated with a very high risk of premature death from all causes, and death from CVD in particular, in patients with IP and RA.

It is interesting to note the similarities in the frequencies of carriage of the PTPN22*1658T susceptibility allele across the different serologic groups, which we subsequently found did not predict mortality. This is in contrast to most previous studies that showed an association between *PTPN22* and anti-CCP antibodies (46,47). The similar frequencies irrespective of anti-CCP status might explain why no association was found for carriage of PTPN22*1858T susceptibility alleles and either all-cause mortality or CVD mortality.

Because this study used a primary care–based cohort of patients with IP, selection bias was limited. Furthermore, the sample size was large, and patients were followed up for long periods of time, and so, we were able to produce robust estimates of the influence of *HLA*–*DRB1* and PTPN22*1858T on the risk of death. For some of the analyses, particularly for the mortality risk estimate between smoking, anti-CCP antibody status, and carriage of 2 copies of SE alleles, the numbers of patients and subsequent deaths were small, and this could result in less-precise estimates. Since the mortality risks were calculated with multivariate models using interaction terms, the potential loss in the robustness of any of the estimates was reduced; furthermore, based on the power calculations, we are confident that this subgroup analysis did not show false-negative results.

Analyses were undertaken in the IP cohort as a whole as well as in the subgroup who met the ACR criteria for RA by 10 years. It could be argued that the start of followup for the RA subgroup should begin at the time of the RA diagnosis (i.e., when they met the ACR criteria). However, we have previously shown that it is difficult to distinguish those who will subsequently have RA by applying the ACR criteria in the early weeks and months following symptom onset (48). Furthermore, while some patients might not have met the ACR criteria for a number of years, we hypothesize that the disease processes that would contribute to the increased mortality risk would begin at symptom onset irrespective of the subsequent RA status. Therefore, followup in all patients began at symptom onset.

A potential limitation of the current study could be the exclusion of patients from the analyses either because they were enrolled in NOAR but did not provide a DNA sample (n = 402) or because they were lost to followup by the ONS (n = 5), but only if these 407 patients were systematically different from the 1,022 patients who were included in the study. However, no systematic differences in clinical characteristics between the two groups were detected.

The serologic markers used in the analyses were obtained at baseline, and it is possible that in patients with early disease, seroconversion may subsequently occur. This is particularly true in the case of RF, and indeed, in the current cohort, 145 patients became seropositive for RF between their baseline and tenth anniversary assessments. However, the purpose of this study was to identify factors at baseline that could be used to predict which patients are at highest risk of subsequent premature death.

In conclusion, we found that *HLA*–*DRB1* genotypes previously associated with vasculitis and extraarticular disease in RA were associated with premature death in patients with IP. This effect was independent of RF and CRP, but an interaction with smoking and anti-CCP antibodies substantially increased the risk of death. This knowledge could facilitate a targeted prevention program for CVD in patients with IP and RA.

## AUTHOR CONTRIBUTIONS

Dr. Farragher and Prof. Symmons had full access to all of the data in the study and take responsibility for the integrity of the data and the accuracy of the data analysis.

**Study design.** Silman, Symmons, Barton.

**Acquisition of data.** Goodson, Naseem, Thomson.

**Analysis and interpretation of data.** Farragher, Silman, Thomson, Symmons, Barton.

**Manuscript preparation.** Farragher, Goodson, Silman, Thomson, Symmons, Barton.

**Statistical analysis.** Farragher, Goodson.
